# Boceprevir-induced Herpes Zoster

**DOI:** 10.5005/jp-journals-10018-1282

**Published:** 2019-02-01

**Authors:** Ayşegul Babalı, Basak Cakal, Alpaslan Tanoglu, Fatih Karaahmet, Murat Kekilli

**Affiliations:** 1 Department of Gastroenterology, Ankara Training and Research Hospital, Ankara, Turkey; 2 Department of Gastroenterology, GATA Haydarpasa Training and Research Hospital, Istanbul, Turkey

**Keywords:** Boceprevir, Herpes zoster, Infection.

## Abstract

Herpes zoster is caused by reactivation of the varicella zoster virus (VZV), that attacks peripheral or cranial nerves and result in painful cutaneous inflammation. Boceprevir is a protease inhibitor which used as a new therapeutic agent for chronic hepatitis C infection. Boceprevir associated herpes zoster is extremely rare condition. We present herpes zoster infection assosiated Boceprevir in patint with chronic hepatitis C.

**How to cite this article:** Babali A, Cakal B, Tanoglu A, Karaahmet F, Kekilli M. Boceprevir-induced Herpes Zoster. Euroasian J Hepatogastroenterol, 2018;8(2):161-162.

## INTRODUCTION

With an estimated of more than 240 million people infected worldwide, chronic hepatitis B (CHB) remain a major public health problem. The CHB is considered a major factor forserious liver diseases such as cirrhosis, hepatocellular carcinoma, and related complications.^1^ Thecurrent antiviral therapies have limited off-treatment efficacy, requiring long-term continuous and expensive treatments and follow-ups; however, definitive viral elimination is infrequent).^2^

Herpes zoster is caused by reactivation of the varicella-zoster virus (VZV), that attacks peripheral or cranial nerves and result in painful cutaneous inflam-mation.^1^ Boceprevir is a protease inhibitor which used as a new therapeutic agent for chronic hepatitis C infec-tion.^2^ Boceprevir has been involved in several articles as a cause of anemia and dysgeusia.^3^ Boceprevir associated herpes zoster is extremely rare condition and has not been reported in individuals.

## CASE REPORT

**Fig. 1:**
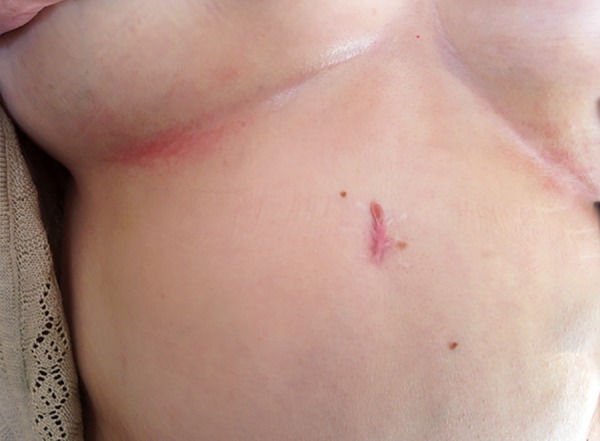
Rash in dermatomal distribution

A 56-year-old woman was presented with an abdominal maculopapular rash with sparing related to herpes zoster. The patient had chronic hepatitis C infection genotype^4^ in May 2013, and she had been on boceprevir treatment for 8 weeks. She had no history of trauma, smoking or drunk and any medications include herbal or illicit drugs. ANA, AMA, Anti Ds SMA levels were all negative. The patient was begun to treat with peginterferon alfa-2b and ribavirin. This combination was ceased since the viral load was not decreased after 12 weeks. In October 2013, boceprevir including triple therapy was started. After using peginterferon alfa-2b and ribavirin for 4 weeks, boceprevir was added to the main treatment. Eight weeks later, in the 12th week, severe back and right side pain occurred. Bright skin rash with blisters was observed under the right breast of the patient (Fig. 1). Routine laboratory tests were within normal range. After consulting with a dermatologist, the case was diagnosed as herpes zoster. At the same time, triple therapy was discontinued because of the high viral load. Antiviral therapy was not prescribed and lesions were healed with symptomatic treatment within few days.

## DISCUSSION

Herpes zoster is caused by reactivation of the varicella-zoster virus (VZV). It causes painful viral skin disease and has only been reported in immunocompromised individuals, such as those with HIV/AIDS, patients receiving immune-suppressive agents such as steroids, radiation, and chemotherapy, or those with a previous history of cancer. The diagnosis Herpes zoster infection is usually based on the characteristic varicella rash, which is vesicular and covers a single dermatome.^4,5^ Boceprevir-induced herpes zoster has not been reported formerly.

Boceprevir is a new protease inhibitor which used to treat chronic hepatitis C infection.^2^ Although no articles have shown boceprevir related herpes zoster or the likely mechanism of causing shingles, it can be theorized that the metabolites of the drug itself or its deposits may trigger the latent varicella zoster virus in the nerve cell bodies. Moreover, boceprevir including triple therapy may weaken the immune system. The Naranjo adverse drug reaction score, which was used to estimate the adverse reaction of the drug, was found.^5^ This score leads to suggest that probable association between boceprevir and Herpes zoster.^6^

Here, we present an extremely rare case of Herpes zoster associated with Boceprevir treatment in chronic hepatitis C patient.
